# Risk of acute brain lesions in dizzy patients presenting to the emergency room: who needs imaging and who does not?

**DOI:** 10.1007/s00415-020-09909-x

**Published:** 2020-05-27

**Authors:** Björn Machner, Jin Hee Choi, Peter Trillenberg, Wolfgang Heide, Christoph Helmchen

**Affiliations:** 1 Department of Neurology, University Hospital Schleswig-Holstein, Campus Lübeck, Ratzeburger Allee 160, 23538 Lübeck, Germany; 2 Department of Neurology, General Hospital Celle, Celle, Germany

**Keywords:** Stroke, Nystagmus, Vertigo, Dizziness, CT, MRI

## Abstract

The usefulness of brain imaging studies in dizzy patients presenting to the emergency department (ED) is controversial. We aimed to assess the ‘real-world’ probability of ischemic stroke and other acute brain lesions (ABLs) in these patients to create an algorithm that helps decision-making on whether which and when brain imaging is needed. By reviewing medical records, we identified 610 patients presenting with dizziness, vertigo or imbalance to our university hospital’s ED and receiving neurological workup. We collected timing/triggers of symptoms, ABCD^2^ score, focal neurological abnormalities, HINTS (head impulse, nystagmus, test-of-skew) and other central oculomotor signs. ABLs were extracted from CT/MRI reports. Uni-/multivariate logistic regression analyses investigated associations between clinical parameters and ABLs. Finally, the likelihood of ABLs was assessed for different clinically defined subgroups (‘dizziness syndromes’). Early CT (day 1) was performed in 539 (88%) and delayed MR imaging (median: day 4) in 299 (49%) patients. ABLs (89% ischemic stroke) were revealed in 75 (24%) of 318 patients with adequate imaging (MRI or lesion-positive CT). The risk for ABLs increased with the presence of central oculomotor signs (odds ratio 2.8, 95% confidence interval 1.5–5.2) or focal abnormalities (OR 3.3, 95% CI 1.8–6.2). The likelihood of ABLs differed between dizziness syndromes, e.g., HINTS-negative acute vestibular syndrome: 0%, acute imbalance syndrome with ABCD^2^-score ≥ 4: 50%. We propose a clinical pathway, according to which patients with HINTS-negative acute vestibular syndrome should not receive brain imaging, whereas imaging is suggested in dizzy patients with acute imbalance, central oculomotor signs or focal abnormalities.

## Introduction

Dizziness is one of the most common presenting complaints in emergency departments (EDs) [17, 30]. Diagnosing patients with dizziness is challenging and ED physicians often request neurological consultation and brain imaging studies to differentiate non-vestibular medical causes (e.g., orthostatic dizziness) from peripheral (e.g., benign paroxysmal positional vertigo) or central vestibular disorders (e.g., brainstem/cerebellar stroke) [11, 27]. The likelihood of detecting an acute brain lesion (ABL), for instance a stroke, inflammatory lesion or tumor, varies greatly depending on the clinical preselection process of patients and the modality and timing of brain imaging studies. Thus, numbers range between 3–4% in unselected cohorts of dizzy patients in the ED [15, 30], 27% in patients with an acute transient vestibular syndrome [3], and up to 75% in clinically preselected cohorts with an acute (persistent) vestibular syndrome and a high-risk vascular profile [14]. Notwithstanding, a stroke is still missed in about 35% of patients presenting with acute dizziness to the ED [15].

Brain imaging studies can help to differentiate peripheral vestibular and central causes of dizziness [13]. However, the sensitivity for detecting an ABL, particularly ischemic stroke, depends on the imaging modality and its timing [33, 34]. Computed tomography (CT) is usually performed in the emergency setting, due to its around-the-clock and on-site availability in most of the hospitals. However, the diagnostic yield of CT brain imaging in the evaluation of non-preselected dizzy patients in the ED is low (~ 2%) [21]. One reason is the CT’s low sensitivity (~ 10%) for acute ischemic strokes [2], especially if they occur in the posterior cranial fossa [33]. In line with that, more CT imaging in the ED does not lead to an improvement in stroke diagnosis [20]. A negative CT result may even falsely reassure the ED physician of a peripheral cause instead of the actual central dizziness [10]. Although magnetic resonance imaging (MRI) has a higher sensitivity for acute ischemic strokes than CT [2], within the first 48 h diffusion-weighted MRI still can be false negative in about 50% of small ischemic strokes in the posterior fossa [34]. Furthermore, MRI is not as available (and feasible) as CT for dizzy patients in the ED and conducting MRIs in all dizzy patients presenting to the ED would unnecessarily burden many patients as well as the health-care system, as it exceeds the acceptable cost limits of most national economies [28, 33].

Hence, there is a clear need of a clinical preselection to identify those patients with a high pre-test probability for an ABL on imaging. However, the sensitivity and specificity of clinical tests that allow differentiation between peripheral and central causes of dizziness, such as the head impulse test [5, 31] or the HINTS triad (head impulse, nystagmus, test of skew) [14, 16], largely depend on the experience of the examiner [18]. On the other hand, ‘rater-independent’ risk scores like the ABCD^2^-score [25] or single risk factors such as high age [26] are less sensitive/specific [32] and they can only provide a rough estimation on the overall probability of a cerebrovascular cause, which may not be helpful in the individual case.

To specify, ED physicians and neurologists, who may not be neuro-otology experts, are confronted on a daily basis with the following questions [6]: Does this dizzy patient needs brain imaging or not? Is a CT sufficient, or is an MRI necessary? Is an immediate imaging study required? How do we deal with a negative result?

We aimed to develop a practical guide based on empirical evidence. By reviewing medical records of more than 600 patients presenting with dizziness, vertigo or imbalance to our university hospitals’ ED, we analyzed the frequency of brain imaging studies and the occurrence of ABLs. Taking established diagnostic algorithms for dizzy ED patients into account [8, 37], we assessed the association of ABLs with specific clinical signs and ‘dizziness syndromes’. Based on the results, we propose a clinical pathway that stratifies dizzy patients into subgroups of those who (i) do not need any brain imaging, (ii) do not need early CT but delayed MRI or (iii) require urgent CT and additional, delayed MRI when necessary.

## Methods

### Study design and setting

For this retrospective, single-center, observational study we reviewed the medical records of patients who presented to the emergency department (ED) of the University of Lübeck Medical Center (Lübeck, Germany). The ED at this tertiary care university hospital encounters about 42,000 patients per year. It is staffed 24/7 with resident and attending internal medicine physicians as well as one neurology resident. There is around-the-clock access to computed tomography (CT) in the ED, whereas MRIs are usually performed electively during daytime.

The study was approved by the Ethics Committee of the University of Lübeck (18-146A).

### Study population

Using the hospital’s medical controlling, we identified all adult (≥ 18 years of age) patients who were (i) presenting to the ED between January 1, 2016 and December 31, 2018, (ii) seen by a neurology resident in the ED and (iii) finally admitted to one of the hospital’s neurological wards (general neurological ward or stroke unit). From these preselected cases, we opened the electronic medical records written by the neurology resident in the ED and searched for any of the following presenting complaints: ‘dizziness’, ‘vertigo’ or ‘imbalance’. By applying these criteria, we included patients with an acute and unclear type of dizziness who required admission to a neurological ward for further diagnostics and treatment. We thereby excluded patients with dizziness due to a clear medical cause and patients with an identified benign paroxysmal positional vertigo (BPPV) who could be successfully treated in the ED and directly discharged home.

For patients with multiple eligible ED visits during the study period, only the first visit was included.

### Baseline measurements

We collected information on patients’ characteristics (age, sex), comorbidities (arterial hypertension, diabetes mellitus, atrial fibrillation, coronary heart disease) and past medical history (prior stroke/TIA, vestibular disorders including Meniere’s disease, vestibular migraine, vestibular neuritis, BPPV). We obtained specific information on the presenting complaint including its character (e.g., ‘spinning’ or ‘swaying’), whether it was persistent (continuously present at the time of presentation in the ED) or transient/episodic, the type of onset (sudden, slowly progressing), the duration and if there were any triggers (e.g., head motion, changes in body position, locomotion) or associated symptoms (nausea/vomiting, headache, hearing disturbances, diplopia, visual field abnormality, speech difficulty, hemi-symptoms including sensory, motor or coordination abnormalities).

From the documentation of the neurological examination, we extracted information on focal abnormalities (aphasia, dysarthria, visual field defects, facial weakness, limb weakness, limb ataxia, sensory impairment) and the HINTS-relevant information including the bedside head impulse test (bHIT), spontaneous nystagmus and test of skew. The HINTS triad was rated as ‘negative’ if (i) the bHIT was abnormal (corrective saccade after horizontal head thrust), (ii) the nystagmus’ fast phase did not change direction with gaze and (iii) there was no skew deviation. HINTS were rated as ‘positive’ if bHIT was documented as normal, the nystagmus’ fast phase alternated with gaze or a skew deviation was observed. We also collected information on the following central oculomotor (OM) signs: horizontal/vertical gaze-evoked nystagmus, vertical or purely torsional spontaneous nystagmus, ophthalmoparesis and disrupted (‘saccadic’) smooth-pursuit eye movements.

The results of the Dix–Hallpike maneuver, if performed, were categorized into ‘BPPV-typical nystagmus’ in the plane of a specific semicircular canal on positional maneuvers, ‘atypical nystagmus’ or ‘no nystagmus’.

Using the blood pressure (RR) documented at ED presentation and parameters mentioned above, we calculated the ABCD^2^ score (range 0–7) for each patient: age 60 years or older = 1; blood pressure ≥ 140/90 = 1; clinical features (unilateral weakness = 2, speech disturbance = 1); duration of symptoms (< 10 min = 0, 10–59 min = 1, ≥ 60 min = 2); and diabetes = 1 [12]. In case of missing information on a specific ABCD^2^ item, this was assigned a score of 1 [25].

### Outcome measures

Brain imaging results (CT, MRI) were abstracted from the official neuroradiological reports. The primary outcome was an ABL on brain imaging including infarction, hemorrhage, intracerebral tumor and inflammatory lesions of the central nervous system (CNS). Residual vascular, post-traumatic or post-interventional defects as well as known and stable tumors (e.g., meningioma) were not attributed as ABL, because they could hardly account for the acute dizziness as a presenting complaint.

CT scans were performed on a 64-slice CT scanner (Siemens SOMATOM Definition AS+) during the patients’ stay in the ED. MRI scans, which always included axial T2 fluid-attenuated inversion recovery (FLAIR) and diffusion-weighted images (DWI) besides other sequences, were obtained on a 1.5 or 3.0 T Philips Achieva MRI scanner, usually some days after admission.

### Statistical analysis

Statistical analyses were performed using SPSS 22.0 (IBM Corp., Somer/NY, US). Descriptive statistics were calculated for all variables of interest, and data are presented as counts and percentages.

Risk factors potentially associated with the primary outcome were assessed via univariate and, if significant, multivariate logistic regression analysis (LRA). Results from the LRA are given as odds ratios (ORs) with 95% confidence interval. *P* values < 0.05 were considered significant.

The frequency of ABLs was calculated separately for the two imaging modalities and for different clinical subgroups of patients. The patient’s assignment to a distinct subgroup was based on recently suggested clinical algorithms for dizzy patients in the ED [8, 37]. The algorithms use information from the patient’s history (timing/trigger of symptoms) and targeted clinical examination (oculomotor signs) to stratify the patient to one specific ‘dizziness syndrome’. These syndromes encompassed the ‘spontaneous transient vestibular syndrome’ (sTVS; vestibular symptoms < 24 h and not present at presentation in the ED, no trigger in the history), ‘triggerable episodic vestibular syndrome’ (tEVS; episodic vestibular symptoms that are provoked by specific triggers, e.g., postural change like in BPPV), ‘acute vestibular syndrome’ (AVS; vestibular symptoms and spontaneous nystagmus, continuously present at ED presentation), and ‘acute imbalance syndrome’ (AIS; acute onset of an unsteadiness in stance and gait, still persistent at ED presentation, no spontaneous nystagmus). Based on the findings from the clinical examination, tEVS patients were further assigned to either a BPPV cohort (typical nystagmus on Dix-Hallpike) or a central positional vertigo (CPV) cohort (atypical or no nystagmus on Dix–Hallpike). Likewise, AVS patients were divided into a HINTS-positive ‘central’ group and a HINTS-negative ‘peripheral’ group. Finally, using the ABCD^2^-score, patients with an AIS were split into a high-risk (≥ 4 points) and a low-risk (< 4 points) AIS subgroup.

## Results

### Demographic, clinical and imaging characteristics

610 patients fulfilled the eligibility criteria and were included in the final analysis. 539 of these patients (88.4%) received a CT in the ED. An MRI was conducted in 299 patients (49.0% of all patients); this usually happened with a delay (median: 4 days) but always within 18 days after admission. Most of the patients with an MRI first received a CT (*n* = 279), i.e., only 20 patients received an MRI without having an initial CT. Only few patients received an MRI on the first day (*n* = 9, 3.0%).

Table 1 provides the demographic and clinical characteristics of the patients, separately for the whole study group and different subgroups (patients with ABLs, patients without ABLs, patients without any imaging).

**Table 1:** Characteristics of the whole study population and different subgroups of patients with respect to the results of the brain imaging studies

Characteristics	All patients (*n* = 610)	Patients with ABL on CT or MRI (*n* = 75)	Patients with no ABL on MRI (*n* = 243)	Patients without brain imaging (*n* = 51)
Age [years; mean ± SD (median)]	65 ± 16 (67)	66 ± 14 (66)	64 ± 15 (64)	61 ± 19 (61)
Female	319 (52)	34 (45)	129 (53)	38 (75)
Comorbidities/vascular risk factors				
Diabetes	85 (14)	15 (20)	32 (13)	2 (4)
Hypertension	318 (52)	49 (65)	121 (50)	20 (39)
Prior stroke	80 (13)	9 (12)	33 (14)	3 (6)
ABCD^2^-score [mean ± SD (median)]	2.9 ± 0.9 (3.0)	3.3 ± 1.2 (3.0)	2.9 ± 0.9 (3.0)	2.7 ± 0.8 (3.0)
ABCD^2^ ≥ 4 (high risk)	121 (20)	27 (36)	42 (17)	9 (18)
Previous diagnosis of a vestibular disorder	88 (14)	5 (7)	38 (16)	18 (35)
Targeted history of the symptom ‘dizziness’				
Vertigo (‘spinning’)	301 (49)	25 (33)	115 (47)	33 (65)
Sudden onset	404 (66)	48 (64)	161 (66)	32 (63)
Episodic	170 (28)	6 (8)	70 (29)	25 (49)
Triggerable	69 (11)	2 (3)	23 (10)	16 (31)
Positional	61 (10)	2 (3)	18 (7)	16 (31)
Associated CNS symptoms	125 (21)	36 (48)	46 (19)	2 (4)
Headache	70 (12)	9 (12)	32 (13)	2 (4)
Hearing disturbance, tinnitus	55 (9)	4 (5)	26 (11)	5 (10)
Findings on clinical examination				
Any central oculomotor sign	124 (20)	33 (44)	41 (17)	4 (8)
Any focal abnormality	136 (22)	41 (55)	51 (21)	3 (6)
Initially admitted to the stroke unit	344 (56)	64 (85)	148 (61)	0 (0)

Data are *n* (%) unless otherwise indicated

*ABL* acute brain lesion

ABLs were identified in 36 of 557 patients (5.6%) receiving early CT and in 56 of 299 patients (18.1%) who received MRI. From the 56 patients with an ABL on MRI, *n* = 17 already had the lesion revealed on the initial CT, *n* = 37 had a normal CT result and *n* = 2 did not have a CT in the ED. There were 19 patients with ABLs detected by the initial CT who did not receive additional MRI. Overall, 75 (23.6%) of 318 dizzy patients who received adequately sensitive brain imaging eventually had an ABL.

The ABLs’ most common etiology was ischemic stroke (*n* = 67, 89.3%), besides two patients with an intracerebral hemorrhage (2.7%), three patients with inflammatory CNS lesions (4.0%), two patients with an intracerebral tumor (2.7%) and one patient with a post-epileptic edema of the hippocampal region.

Patients who did not receive any brain imaging (Table 1, last column) were mostly female (75%) and often reported transient dizziness symptoms (49%) and positional changes as trigger (31%). Many of them had a previous history of vestibular disorders (35%) and only few exhibited central OM signs (8%) or focal abnormalities (6%) on clinical examination.

### Clinical predictors for ABLs

To identify clinical markers predicting an ‘ABL’ (dependent variable), odds ratios were calculated for several clinical parameters by using univariate/multivariate logistic regression analyses (Table 2).

**Table 2:** Clinical predictors for an acute brain lesion in 318 patients presenting with dizziness to the emergency department

Parameters	Univariate analysis		Multivariate analysis	
	OR (95% CI)	*p* value	OR (95% CI)	*p* value
Vascular risk profile				
Age ≥ 60 years	1.2 (0.7–2.0)	0.603		
Arterial hypertension	**1.9 (1.1–3.3)**	**0.019**	1.6 (0.9–3.0)	0.103
Diabetes	1.7 (0.9–3.3)	0.148		
Prior stroke	0.9 (0.4–1.9)	0.724		
ABCD^2^-score ≥ 4 (‘high risk’)	**2.7 (1.5–4.8)**	**0.001**	1.5 (0.8–3.0)	0.245
Symptomatology				
Vertigo (‘spinning’)	**0.6 (0.3–0.9)**	**0.034**	0.6 (0.3–1.1)	0.127
Sudden onset	0.9 (0.5–1.6)	0.719		
Transient symptoms	**0.2 (0.1–0.5)**	**0.001**	**0.3 (0.1–0.6)**	**0.004**
Positional change as trigger	0.3 (0.1–1.5)	0.157		
Hearing disturbance	0.5 (0.2–1.4)	0.173		
Headache	0.9 (0.4–2.0)	0.792		
Associated CNS symptoms	1.0 (1.0–1.0)	0.058		
Clinical examination				
Any central oculomotor sign	**3.9 (2.2–6.8)**	**< 0.001**	**2.8 (1.5–5.2)**	**0.001**
Any focal abnormality	**4.5 (2.6–7.9)**	**< 0.001**	**3.3 (1.8–6.2)**	**< 0.001**

Only patients with sufficiently sensitive brain imaging (MRI or lesion-positive CT) were included in this analysis, which applied to 318 of 610 patients

Univariate analyses revealed that ABLs were more likely in patients with (i) preexisting arterial hypertension, (ii) high-risk ABCD^2^ score ≥ 4, (iii) any central OM sign and (iv) any focal abnormality on clinical examination. In contrast, ABLs were found to be less likely in (v) patients who reported ‘vertigo with a sense of spinning’ as the dizziness’ character and (vi) those with transient dizziness symptoms.

In the multivariate model, only central OM signs (OR 2.8, 95% CI 1.5–5.2) and focal abnormalities on clinical examination (OR 3.3, 95% CI 1.8–6.2) remained statistically significantly positive predictors for ABLs, while the transient nature of symptoms was associated with a reduced risk for ABLs (OR 0.3, 95% CI 0.1–0.6).

### Occurrence of ABLs within clinically defined patient subgroups (dizziness syndromes)

**Fig. 1:**
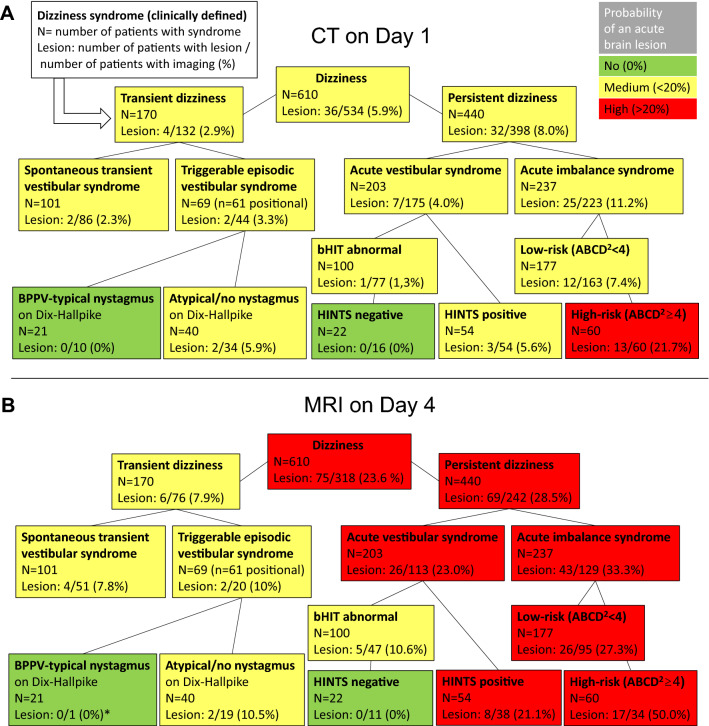
Probability of an acute brain lesion as detected by early CT **a** or delayed MR imaging **b** in dependence of the patients’ clinical sub-specification (‘dizziness syndrome’). For the purpose of clarity, we color coded the probability/risk to have an acute brain lesion (ABL) revealed by the respective imaging study (green: no risk, yellow: low–medium risk, red: high risk). **a** Early CT imaging performed in 534 of 610 ‘dizzy’ patients revealed ABLs in 36 of them (5.9%). Further stratification of the patients by using information from targeted history taking and clinical examination can increase the probability of detecting ABLs on CT to over 20% (e.g., ‘acute imbalance syndrome (AIS) with high-risk ABCD^2^-score’). **b** Delayed MRI is more sensitive in detecting ABLs and identifies high-risk subgroups (e.g., ABLs in 50% of AIS patients with ABCD^2^ ≥ 4), but also no-risk subgroups (e.g., 0% ABLs in HINTS-negative AVS patients). Notably, **b** also includes those patients with an ABL already detected on the early CT (‘lesion-positive’ CT) who did not receive a redundant MRI. *Patients with benign paroxysmal positional vertigo (BPPV) were generally rare in our study cohort as they were usually identified and directly discharged from the ED

We analyzed the frequency of patients who received brain imaging and the number of cases in which an ABL was detected, separately for the two imaging modalities and the different clinically defined subgroups (see “Methods”). The probability of detecting a lesion was greater with MR (Fig. 1b) than CT imaging (Fig. 1a), and more importantly, the clinical subgroups were associated with a very different risk of ABLs.

There were ‘zero-risk’ subgroups including patients with episodic vertigo triggered by positional change and typical nystagmus on Dix–Hallpike (BPPV group), whereas similarly presenting patients with no or an atypical nystagmus, indicating central positional vertigo (CPV group), revealed ABLs in 6% of CT and 11% of MRI scans.

Not a single patient with an acute vestibular syndrome (AVS), in whom the complete HINTS triad was negative, revealed ABLs. MRIs disclosed ABLs in 11% of those AVS patients with an abnormal bHIT, but without the other two HINTS items (Fig. 1b).

The ABL risk was highest in patients with an ‘acute imbalance syndrome (AIS)’, defined by persistent dizziness and imbalance but no nystagmus. The likelihood of detecting an ABL was 11% on the initial CT, increasing to 33% on the delayed MRI. AIS patients with a high-risk ABCD^2^-score ≥ 4 even revealed ABLs in 50% of MRIs (Fig. 1b).

The probability of detecting an ABL in patients with ‘spontaneous transient vestibular syndrome (sTVS)’ was very low on early CT imaging (2%) and only moderate on delayed MRI (8%). Separate analysis of high-risk sTVS patients with an ABCD^2^-score ≥ 4 and/or focal CNS symptoms during the attack (*n* = 31) did not increase the specificity, i.e., their probability of ABLs on MRI (2/20, 10%) was almost as high as in the whole sTVS group. Accordingly, two patients with a low-risk ABCD^2^-score < 4 and no history of focal CNS symptoms still revealed ABLs (2/31, 6.5%).

## Discussion

We analyzed the ‘real-world’ frequency and diagnostic yield of brain imaging studies (CT, MRI) in patients presenting with dizziness, vertigo or imbalance to the ED, excluding those with a clear medical cause or uncomplicated BPPV. Early CT imaging was conducted in over 80% of the patients and almost every second patient later received an MRI (median 4 days). Dizzy patients who did not receive brain imaging were mostly female, typically reported transient/triggerable symptoms and seldom exhibited central oculomotor signs or other focal abnormalities on clinical examination. This most probably reflects a preexisting selection strategy in our ED to especially prevent young women with transient symptoms and normal neurological examination from unnecessary CT imaging.

Every fourth patient (24%) revealed an ABL (usually ischemic stroke) on adequately sensitive brain imaging. This could be taken as an argument to perform brain imaging in every dizzy patient presenting to the ED. However, in light of the high economic burden and overexposure of patients to imaging studies, we should make use of clinical markers that allow to preselect those dizzy patients who have a high pre-test probability for a central lesion.

In our cohort of dizzy ED patients, we found two clinical markers to be independent predictors for an ABL: any central oculomotor sign or a focal abnormality detected on neurological examination. In contrast, vascular risk factors such as arterial hypertension or an ABCD^2^-score ≥ 4 were only associated with ABLs in the univariate, but not in the multivariate logistic regression analysis. These results are in line with the previously proposed markers for central causes of acute dizziness: Navi and colleagues identified ‘focal examination abnormality’, ‘higher age (≥ 60 years)’ and ‘imbalance as presenting complaint’ as independent predictors [26]. Kerber et al. found the ‘continuous ABCD^2^ score’, a ‘central pattern on the oculomotor assessment’, ‘any other CNS feature’ on clinical examination and ‘prior stroke’ to be related with stroke in patients with ‘new and continuous dizziness’ [16]. Taken together, brain imaging is strongly suggested in dizzy patients who exhibit any central oculomotor sign or focal abnormality on neurological examination. In contrast, vascular risk factors such as higher age or an ABCD^2^-score ≥ 4 are not consistently found to be reliable and independent predictors for ABLs in dizzy patients and should therefore not solely trigger imaging studies.

But how shall we manage dizzy patients in the ED who do not reveal any central oculomotor sign or focal abnormality? Conducting brain imaging in those with at least one vascular risk factor would lead to an excessive use of imaging studies in many dizzy patients with an actually benign peripheral-vestibular or non-vestibular cause. On the other hand, applying risk factors as ‘conditio sine qua non’ may prevent necessary imaging studies in juvenile stroke patients with a dissection or thromboembolic occlusion of the vertebral artery [19].

**Fig. 2:**
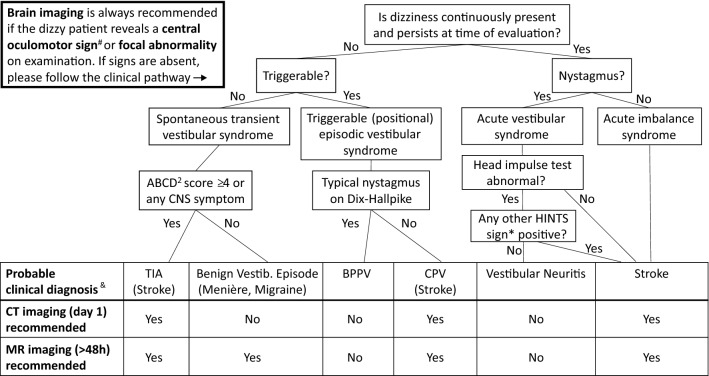
Clinical pathway to help decision-making on brain imaging in patients presenting to the ED with dizziness, vertigo or imbalance and without a general medical cause^+^. *TIA* transient ischemic attack, *BPPV* benign paroxysmal positional vertigo, *CPV* central positional vertigo. ^+^General medical causes comprise various toxic, metabolic, infectious, or cardiovascular diseases (see Edlow et al. 2018 [8]). ^#^Central oculomotor signs include: vertical or purely torsional spontaneous nystagmus, horizontal/vertical gaze-evoked nystagmus, gaze palsies, bilaterally disrupted smooth pursuit eye movements.*HINTS are positive (‘central’) if any of the following signs is present: normal head impulse test, the nystagmus’ fast phase alternating with gaze, skew deviation with a refixation on cover test. ^§^The MRI may be dispensable if the lesion has already been detected by early CT. ^&^Only the most likely and most relevant differential diagnosis is stated.

We rather suggest a clinical stratification of patients into specific ‘dizziness syndromes’, based on symptoms (timing, triggers) and signs from a targeted clinical examination [8, 37]. We could show that these previously proposed (and slightly modified) algorithms can be successfully applied to clinical routine in the ED when dizzy patients are assessed by non-experts in neuro-otology. Using their clinical assessment allowed to distinguish dizzy patients with a zero, moderate or high pre-test probability for ABLs on brain imaging. Based on that, we developed a clinical pathway (Fig. 2) that can be used practically by clinicians working in EDs to decide whether an individual dizzy patient should receive brain imaging or not.

To specify, patients with an episodic vestibular syndrome triggered by positional change should only receive brain imaging when there is no BPPV-typical nystagmus on Dix–Hallpike maneuver and CPV is suspected. In AVS patients, brain imaging is dispensable if the HINTS-triad is negative. While the HINTS’ high sensitivity for detecting strokes in dizzy patients is known to neuro-oto-/ophthalmological experts [14, 32], our study impressively confirms their usefulness when applied by neurology residents under ‘real-world’ conditions in the ED. When the bHIT is used as a single test to differentiate peripheral and central vestibular causes in AVS, neurology residents even revealed a higher sensitivity for central pathologies than experts in previous studies: only 11% of patients with an abnormal bHIT (indicating peripheral-vestibular pathology) later disclosed strokes on MRI versus 30% of patients in studies using experts’ assessments [5, 31]. We like to emphasize, however, that we definitely recommend using the complete HINTS triad, or even the HINTS+ assessment (including examination of a sudden hearing loss) [32], because the bHIT as a single test may be abnormal (and thereby falsely indicate an isolated peripheral vestibulopathy) in some patients with circumscribed ponto-cerebellar lesions or a combined peripheral–central vestibulopathy as in AICA infarctions that include the labyrinthine artery [4, 22, 31]. AVS patients with a normal bHIT or any other positive HINTS sign (skew deviation, nystagmus’ fast phase alternating, sudden hearing loss) should always receive brain imaging as previously suggested [14, 32] and confirmed in our cohort (21% ABLs).

Patients with acute imbalance exhibited the highest risk for ABLs among all dizziness syndromes (ABLs in 50% of vascular high-risk AIS patients). Even AIS patients with a ‘low risk’ ABCD^2^-score < 4 had a considerable risk of ABLs (27%). Therefore, we would extend the recommendation by Zwergal et al. [37] that brain imaging should be performed in all AIS patients independent of their individual vascular risk profile.

Patients with spontaneous transient vestibular syndrome (sTVS) remain the most challenging subgroup. Differential diagnoses encompass vertebrobasilar transient ischemic attacks (TIA), (first) manifestation of a ‘benign’ episodic vestibular disease (Meniere’s disease, vestibular migraine) and also non-vestibular causes such as transient disturbances of blood glucose/electrolytes, cardiovascular disorders (arrhythmia) and attacks of phobic/functional vertigo [29]. Clinical examination can hardly contribute to making the diagnosis, as it is usually normal when the asymptomatic patient is assessed in the ED, and transient (vestibular) symptoms are a proven risk factor for missing a stroke in the ED [36]. Brain imaging may be helpful, since diffusion-weighted MRI has been shown to detect acute ischemic lesions in 34% of TIA patients [1] and in 15% of sTVS patients [3]. In our cohort, only 8% of sTVS patients revealed an ABL on MRI. However, absence of an MRI-DWI lesion does not rule out a transient ischemia [3]. Although we could not relevantly increase the probability of detecting ABLs when including the ABCD^2^-score and CNS symptoms during the attack into our model, as a practical approach we suggest to consider brain imaging in those sTVS patients with an ABCD^2^-score ≥ 4 or accessory CNS symptoms. If any of these features is present, early brain imaging is recommended and, in case of sudden head or neck pain potentially indicating vascular dissection, an additional angiogram [9]. If markers are negative and this was the first vestibular episode, we would refrain from early CT but recommend a delayed MRI during the stay in the hospital. In case of recurrent episodes (most probable differential diagnoses: vestibular migraine, Meniere’s disease), a non-urgent MRI should be performed once to exclude rare structural pathologies such as vestibular schwannoma [23] or a pathological contact between the eighth nerve and a cerebellar artery as in vestibular paroxysmia [35].

### Which imaging modality (CT or MRI) and when?

In an ideal world, dizzy patients with clinical indicators of a central cause would primarily receive MRI because it has less radiation risks than CT and higher sensitivity for ischemic stroke and other CNS pathologies [13, 29]. Accordingly, in our study, an ABL was revealed in only 5.6% of the patients who received an (early) CT versus 18.1% of patients who received a (delayed) MRI. Of course, this difference is also influenced by the temporal order effect (first CT, second MRI), as the likelihood to detect an acute stroke lesion is generally greater > 48 h after symptom onset than on the first day.

However, most of the hospitals (even at tertiary medical centers in highly developed countries) have to deal with infrastructural restrictions such as a reduced number of available MRI slots and gaps in the around-the-clock availability of MRI-experienced staff. Moreover, MRI in the acute stage of vestibular disorders may sometimes not be feasible if the patient suffers from severe nausea and vomiting. Furthermore, there are time-critical situations where the imaging study must be conducted immediately, e.g., before thrombolysis in acute ischemic strokes. We therefore regard it more realistic to (i) preselect patients who require brain imaging according to our proposed clinical pathway, (ii) perform an early CT in the ED and (iii) add a delayed MRI when necessary. Performing the MRI with a temporal delay also reduces the risk of missing small vertebrobasilar infarcts due to false-negative DWI scans in the first 48 h [14, 34]. Another advantage is that the CT is sensitive enough to rule out an intracranial hemorrhage and that patients with suggested ischemic stroke can immediately receive antiplatelet medication (or anticoagulants under specific conditions) before the MRI is conducted 3 days later.

Notably, a lack of an ABL even on delayed MRI does not completely rule out a central etiology of the dizziness. First, there is the possibility of small MRI-negative brainstem strokes, which were estimated to have a prevalence between 6.8% [7] and up to 29% [24]. Second, the CNS disorder may have been transient (e.g., TIA or vestibular migraine) and did not cause an ABL. On the other hand, signs or symptoms that were clinically suspected to be due to a central etiology may not always indicate true and acute CNS lesions, e.g., a mild dysmetria in elderly patients interpreted as limb ataxia or sensory symptoms during the vestibular attack which may also be due to a functional/phobic etiology.

## Limitations

In contrast to a prospective controlled study, this study has some limitations that are implicitly due to its retrospective design and the data acquisition in a real-world setting. First, there may have been a selection bias during the initial assessment in the ED when deciding which patient needs to be admitted to the neurological service and which patient should receive brain imaging. Indeed, we cannot fully exclude that patients who did not receive any brain imaging or only a CT scan would have shown an acute brain lesion on proper MR imaging. Furthermore, patients were examined by different neurology residents with different levels of experience and they all followed a certain clinical routine but they did not fill out a structured study protocol, e.g., when assessing the HINTS and other clinical signs. Hence, not all items/variables were available for all the individual patients and the final assessment of the signs depended on the individual level of experience and skills of the examiner.

Furthermore, we cannot rule out that the main outcome measure of this study—an ABL usually defined by a DWI lesion on delayed MRI—may not have missed a few ischemic strokes that were small enough to be MRI negative [7]. However, due to the retrospective design of the study under real life but uncontrolled circumstances, we regarded a DWI lesion on delayed MR imaging to be a more reliable marker for the final diagnosis of a stroke than the clinical diagnosis that may have been solely based on the clinical assessment (e.g., the HINTS examination) performed by different clinicians with varying levels of experience.

## Conclusion

Patients presenting with dizziness, vertigo or imbalance to the ED should first be screened for a general medical cause [8, 30] as well as BPPV as a common and easily treatable disorder. Next, thorough history taking should include timing and triggers of dizziness symptoms and clinical examination should focus on central oculomotor signs and focal abnormalities indicating CNS involvement. Patients with any of the latter signs should undergo brain imaging, usually an early CT in the ED and a delayed MRI after admission. In the remaining patients, decision-making on the need, modality and timing of imaging studies can by guided by our proposed clinical pathway which integrates the empiric risk for ABLs into established clinical algorithms for dizzy patients. Using this practical approach could prevent many unnecessary brain imaging studies in dizzy patients without a central cause and assure imaging in those who require it.

## Data Availability

The data that support the findings of this study are available from the corresponding author upon reasonable request.
